# 

**Published:** 1852-10

**Authors:** 


### CONTENTS OF VOLUME VI.

American Dental Patents. By Geo. W. Kendall.................................. 245
American Society of Dental Surgeons.............................................. 56
A new method of supplying Artificial Teeth and Gums. By W. M. Hunter.......... 14
Advertising ................................................................ 58
A new mode of Treating Exposed Dental Nerves.................................. 57
Arrearages for the Register.................................................. 58
Agents...................................................................... 58
Address of Dr Taft, before the Mississippi Valley Association of Dental Sargeons, February 17th, 1853.................................................... 119
A Method of forming Sockets for Gum Teeth on Plates............................ 127
Answer to Dr. Drakes Interrogatories, continued.............................. 133, 188
A case of Third Dentition. By J. W. Keys, M. D., D. D. S........................ 152
A case of Reproduction of a Portion of the Lower Jaw............................ 153
A few Weeks Abroad.......................................................... 167
Artificial Lips and Noses.................................................... 238
Blow Pipe, By J. B. Williams................................................ 219
Cases of Intermittent Odontangle or Neuralgia. By Dr. H. B. Smith............... 218
Chase on Making Metalic Casts, &c............................................ 50
Dr. Taft's Address before the Mississippi Valley Association of Dental Surgeons.... 6
Dr. Drakes Interrogatories................................................... 59
Dentists' Fees. By Dr. C. P. Culver.......................................... 91
Dr. Morton vs. Dr. Jackson—Sulphuric Ether..................................... 94
Dental Expositor,............................................................ 114
Dr. B. A. Satterthwaite, Lima, O............................................. 114
Dr. J. Harris on Springing of Plates,........................................ 116
Drs. Allen and Hunter—Priority of Improvement.................................. 116
Dental Furnishing Establishment............................................. 117
Deep-seated Dental Caries. By W. H. Dwinelle,................................ 229
Death of Prof. Drake......................................................... 118
Death of Drs. Stipp and Cleveland,........................................... 118
Dr. Watt's Address before the Mississippi Valley Association of Dental Surgeons.. 179
Dr. Hamell's " " " " " " " 184
Excission of the Maxillary Bone. By S. D. Gross,.............................. 30
Extraction of Teeth. By Dr. J. Taylor................................ 84, 155, 199
Examination of the Teeth..................................................... 225
Filling Teeth over exposed Nerves. By Dr. S. P. Miller,........................ 107
Filling Teeth. By Dr. Clark,................................................. 79
Horned Woman................................................................ 242
Illustrated Magazine of Art,................................................. 116
Impracticable Theories,...................................................... 239
John D. Chevalier............................................................ 250

### *Contents.*

Journals received........................................................ 250
Letter from Dr. J. Murray,................................................ 53
Making Gold Plate and Solder,.............................................. 56
Mississippi Valley Association of Dental Surgeons,........................ 55, 116
Minutes of the Second Annual Meeting of the Ohio Dental College Association,...... 142
Muse on Artificial Teeth,.................................................. 154
New Dental Works,.......................................................... 114
Ninth Annual Meeting of the Mississippi Valley Association of Dental Surgeons,.... 145
New Styptic................................................................ 241
Ohio College of Dental Surgery,............................................ 55, 176
Our Dental Abstracts....................................................... 248
Physicians Extracting Teeth,............................................... 129
Proceedings of the Mississippi Valley Association of Dental Surgeons,............. 1
Pittsburg Dentists.......................................................... 56
Prof. Harris' Principles and Practice of Dental Surgery,................... 113
Porcelain Teeth,........................................................... 114
Postage.................................................................... 249
Present Number of the Register............................................. 248
Practical Hints on the Teeth............................................... 249
Prize Essays............................................................... 117, 176
Philadelphia College of Dental Surgery,.................................... 176
Plate Teeth. By J. S. Clark................................................ 209
Report of the Annual Meeting of the American Society of Dental Surgeons........... 25
Remarks on Making Gold Plate and Solder.................................... 47
Risodontrypy or Hullihens operation........................................ 113
" By Dr. Cone.............................................................. 160, 221
" By the Editor,............................................................ 174
Recipe..................................................................... 187
Removal of Teeth and Fangs. By J. S. Clark................................. 212
Report of the Organization of the Dental Association of Allegheny county, Pa...... 243
Springing of Plates, By Dr. Taylor......................................... 10
" " By Dr. J. Harris....................................................... 83
" " By S. D. Muse........................................................... 196
Subscribers................................................................ 249
Sad Casuality.............................................................. 249
Stockholders of the Ohio College of Dental Surgery,........................ 250
Sources of Vitality of the Teeth........................................... 247
Specimens of Artificial Teeth.............................................. 248
The Register............................................................... 57
To the Profession.......................................................... 57
The Southern Journal of the Medical and Physical Sciences.................. 114, 177
The Ohio College of Dental Surgery......................................... 116
Thompson's Compound Self-adjusting Blow-pipe............................... 250
The Commencement Exercises of the Ohio College of Dental Surgeons........... 185
The April Number of the Dental News Letter................................. 234
World's Fair............................................................... 115

### Editorial.

### MISSISSIPPI VALLEY ASSOCIATION OF DENTAL SURGEONS.

It will be seen by reference to the proceedings of this society, that it has changed its time of meeting; several reasons made this desirable. The past few years the cholera has been more or less in the city about the time of the meeting. This with the low stage of water which generally prevails at that season has done much to prevent a general attendance. It is a season too, when those in the city feel most disposed to be absent. We hope the former of these reasons may not again prevail. Yet the low stage of water, and the disposition to take a little relaxation from the arduous duties of the profession, will perhaps always be the same, so that we think the change one calculated to benefit the society. The meeting will always take place hereafter, immediately before or after the commencement of the Ohio College of Dental Surgery, and we hope that this will give some additional interest to the occasion. The 19th and 20th of February is appropriated to the examination of the graduating class, and the commencement exercises of the College; and the Mississippi Association will commence on the 17th and 18th, and we hope the members will be induced to stay, and witness the closing exercises of the institution.

Owing to the few in attendance at the recent meeting of the society—but little was done—yet we hope the spirit which prevailed will awaken new interest, and that the society will long exert an influence for good on the profession.

### THE OHIO DENTAL COLLEGE ASSOCIATION.

We hope that the members of this Association will bear in mind the 19th of February, as the time for the annual meeting of the society. There will be much business, of an interesting character, to attend to. A code of By-Laws will be submitted for adoption; two or three essays read, and disscussions on general subjects, of a professional character.

All graduates of the Institution are eligible to membership, also stock-holders. It is hoped all such will be in attendance.

---

### PITTSBURG DENTISTS.

We have been informed that the Profession, in Pittsburg, have formed an Association for mutual improvement. We are glad to see this spirit, and if all will enter into it with the right feeling, much good will result. By such Associations useful knowledge is disseminated, and that selfish spirit, which looks bad and is bad, is subdued, and a spirit of sociability engendered.

---

### THE AMERICAN SOCIETY OF DENTAL SURGEONS.

The proceedings of this Society, at its last meeting, have not reached us, save the short report which we published. We presume that much of interest transpired.

---

### MAKING GOLD PLATE AND SOLDER.

### A NEW MODE OF TREATING EXPOSED DENTAL NERVES.

### THE REGISTER.

The present number has been delayed beyond the usual time, which we hope will not be the case soon again. This has been owing to new arrangements for publishing, and pressing engagements of other matters which needed our close attention for the last few weeks.

We would be glad if our subscribers would more generally adop the system of advance payment. They thereby would save the postage, and we should be far better enabled to conduct it to their satisfaction. We are satisfied, that it is with most nothing but neglect that it is not done.

We have on hand a few set of the back numbers which will we furnish to new subscribers, who will pay for volume six, in advance, at the rate of $1,50, per volume.

---

### TO THE PROFESSION.

Those who receive the present number of the Register, and who have not, heretofore, been subscribers, will be considered as such, unless it is returned, or we are notified, by letter, that they do not wish to take it. If returned, please direct, Dental Register, Cincinnati, Ohio.

### ADVERTISING.

We would remind those engaged in supplying the Profession with teeth, instruments, &c., &c., that we keep an advertising sheet for their benefit, as well as our own, and we shall be glad to receive their orders.

The price for advertising is, for twelve lines or less, five dollars, per year; longer advertisements in proportion.

### ARREARAGES FOR THE REGISTER.

Subscribers are informed that the dues for the Register are payable to the Editor, and as he has assumed the liabilities of ǝɥʇ paper, he hopes they will send the needful to meet them.

### AGENTS.

On the first page of the cover we have given a list of our agents, the receipts of whom will be good at any time. If Ohio, Indiana, Kentucky, or Eastern funds cannot easily be procured, any par funds, where subscribers reside, will be received for the Register. Remittances may be made at our risk.

### RECEIPTS FOR VOL. 5. DENTAL REGISTER.

A. Berry, Raymond, Miss.
W. B. Ross, Newport, Ky.
J. Taft, Xenia, Ohio.
W. R. Scott, Raleigh, N. C.
T. B. Hamlin, Nashville, Tenn.
W. S. Chandler, Port Gibson, Miss.
Horatio N. Fenn, Rochester, N. Y.
A. M. Leslie, Cincinnati, O.
W. M. Wright, Pittsburgh, Pa.
H. McCullum, Augusta, Ky.
S. D'Muse, Sharon, Miss.
W. H. Goddard, Louisville, Ky.
Charles Bonsall, Cincinnati, O.
George Watt, Xenia, O.
J. G. Hamill, Ripley, O,
John McClure, Carrolton, Miss. $1,00
E. Bray, Evansville, Ia.
J. Forbes, St. Louis, Mo.
J. R. Davis, Rosco, P. O. Ky.
Charles Roberts, Poughkeepsie, N. Y.
P. G. C. Hunt, Indianapolis, Ia.
W. A. Pease, Dayton, O.
Thos. H. Brewster, Bellbrook, O.
I. N. Keely, Brookville, Ia.
Samuel B. Cutter, Holly Springs, Miss.
S. Clippinger, Rossville, Ohio.
T. P. Lucas, Bloomington, Ia.
S. P. Hulihen, Wheeling, Va.

### RECEIPTS FOR VOL. 6. DENTAL REGISTER.

J. A. Weaver, Batavia, O.
Samuel Griffith, Louisville, Ky.
H. R. Smith, Terra Haute, Ia.
Wm. M. Wright, Pittsburgh, Pa. $1.
Merchant Kelly, Bentonville, Ia.
J. M. Cochran, Bastrop, Texas.
James B. Streeter, Allegan, Mich.
J. Murry, Beaver, Pa.
Charles S. Lee, Elkhart, Ia.
T. R. Willard, Dayton, O.

EIGHTH ANNUAL ANNOUNCEMENT OF

### THE OHIO COLLEGE OF DENTAL SURGERY.

**SESSION OF 1852--'53.**

---

The regular course of Lectures in this Institution commences the first Monday of November and closes on the 20th of February.

### FACULTY.

JAMES TAYLOR, M.D., D.D.S.,
*Professor of the Principles and Practice of Dental Surgery.*

THOMAS WOOD, M. D.
*Professor of Anatomy and Physiology.*

GEORGE MENDENHALL, M. D.
*Professor of Pathology and Therapeutics.*

JOHN ALLEN, D.D.S.
*Professor of Operative and Mechanical Dentistry.*

G. L. VAN EMON, A.M. D.D.S.,
*Lecturer on Dental Pharmacy and Demonstrator of Operative and Mechanical Dentistry.*

### EXAMINING COMMITTEE.—DENTAL.

Dr. R. SOMERBY, Louisville, Ky.,
" E. HALE, St. Louis, Mo.,
" A. L. TALBERT, Dayton, Ohio.

### MEDICAL.

Dr. P. S. CONNER, Cincinnati, Ohio.
" P. G. FORE, " "

The Faculty, in issuing this their eighth annual announcement, take pleasure in stating that the Institution is established on a permanent basis, and is in a flourishing condition. They feel that the difficulties which present themselves in the establishment of a new Institution of the kind, have been overcome, and that now a systematic course of instruction has been obtained which shall meet the wants and necessities of the profession.

A building, suitable in every respect for the purposes of a Dental College, is now owned by the profession and dedicated to the advancement of Dental Science.

A fund has been appropriated for the purchase of a Library, and such apparatus as may from time to time appear necessary.

The arrangements in the Laboratory are such as to afford every facility in Mechanical Dentistry, and a demonstrator of experience and ability takes charge of this department.

| | |
|---|---|
| Tickets for the entire course, | $100 00 |
| Matriculating Ticket, | 5 00 |
| Diploma fee, | 25,00 |

Good board can be had from $2 to $3 per week.

JAMES TAYLOR, *Dean.*

### PROSPECTUS

OF THE

### DENTAL REGISTER OF THE WEST.

The Register has now completed its fifth volume, and it will be seen by a resolution of the Mississippi Valley Association, that the work has been given into our hands for future management.

The question has been now practically solved that such a work can be sustained by the Profession, and we presume that no one will doubt but that a work of the kind is needed.

Believing that the interest of the Profession in the West, demand a channel for the dissemination of useful knowledge. We have determined to continue the publication of the Register, and will now say to the Profession that it will be made as valuable in reading matter, and as neat in mechanical execution, as their liberality will possibly justify. There are two things absolutely essential to make it such a work as we should like to see in the hands of the Profession. The first is money to "pay the printer" and the other (which is equally important), well written articles for its pages. The former we are satisfied, is in the hands of the Profession, and if they deliberately view the condition of Dental Science, the progress that is being made, and the need of periodical publications, to keep pace with the improvements of the age, and to collect and preserve such for future use, will be cheerfully furnished. If the Profession East, with the aid of the West, sustain four such publications? should not the West with the aid of the East sustain one?

In general enterprise—in ambition to excel, and all the elements for the advance of Dental Science—we are not behind as we believe any portion of the world, and should not this be developed, and made to tell on this land of future renown? We think it should.

The latter, which is all important, and without which, all the gold in California would be of no avail, we believe, if not in the hands, is in the *heads* of the Profession. There is talent, which if properly exercised would do much to elevate Dental Science. Many of our practitioners are doing but little to make known the result of their experience. They should bear in mind that what to them appears old and common, may be new and of interest to others.

The columns of the Register shall always be open to the profession, for the publication of well written practical or theoretical matter which may be of general interest. We shall, however, aim to keep its columns clear of all which is of a party, personal, or abusive character.

The size of the Register, style of execution, &c. will be much the same as the last volume, and will be published quarterly, at $2 per annum; to those paying in advance, postage free. JAMES TAYLOR.

